# MiR-101 reverses the hypomethylation of the LMO3 promoter in glioma cells

**DOI:** 10.18632/oncotarget.3181

**Published:** 2015-02-11

**Authors:** Xiaoping Liu, Qianqian Lei, Zhibin Yu, Gang Xu, Hailin Tang, Wei Wang, Zeyou Wang, Guiyuan Li, Minghua Wu

**Affiliations:** ^1^ Hunan Provincial Tumor Hospital and the Affiliated Tumor Hospital of Xiangya Medical School, Central South University, Changsha 410013, Hunan, China; ^2^ Department of Breast Oncology, Sun Yat-Sen University Cancer Center, State Key Laboratory of Oncology in South China, Collaborative Innovation Center for Cancer Medicine, Guangzhou 510060, Guangdong, China; ^3^ School of Basic Medical Science, Cancer Research Institute, Central South University, Key Laboratory of Carcinogenesis and Cancer Invasion, Ministry of Education, Key Laboratory of Carcinogenesis, Ministry of Health, Changsha 410078, Hunan, China; ^4^ Medical College, University of South China, Hengyang 421001, Hunan, China

**Keywords:** Epigenetics, MiR-101, LIM-only protein 3, Apoptosis, Polycomb Repressive Complex 2

## Abstract

LIM-only protein 3 (LMO3), a member of the LIM-only protein group, is a new DNA methylation gene that was identified in gliomas via the MeDIP-Chip in our previous study. In this study, we found that LIM-only protein 3 (LMO3) is hypomethylated and overexpressed in glioma cells and tissues. The overexpression of LMO3 was correlated with a poor prognosis in glioma patients, and LMO3 was indirectly inhibited by the tumor suppressor miR-101, which is a potential prognosis marker of gliomas. MiR-101 decreased the expression of LMO3 by reversing the methylation status of the LMO3 promoter and by inhibiting the presence of the methylation-related histones H3K4me2 and H3K27me3 and increasing the presence of H3K9me3 and H4K20me3 on the promoter. It was determined that miR-101 decreases the occupancy of H3K27me3 by inhibiting EZH2, DNMT3A and EED and decreases the H3K9me3 occupancy on the LMO3 promoter via SUV39H1, SUV39H2, G9a and PHF8. Furthermore, miR-101 suppresses the expression of LMO3 by decreasing USF and MZF1.

## INTRODUCTION

Astrocytomas are the most frequently occurring primary brain tumor in adults. Glioblastomas, the most common astrocytoma histology, have a 5-year relative survival of 5% [1]. The median survival time following the diagnosis of a glioblastoma is 15 months [2], and a successful treatment for high-grade astrocytoma is currently lacking. Therefore, there is an urgent need to develop mechanism-based approaches for the management of astrocytomas.

In a previous study, we constructed the DNA methylome in gliomas using high-throughput methylated DNA IP combined with the use of a promoter and CpG island microarrays (MeDIP-Chip) [3]. We then verified three new hypomethylated genes, F10 [4], POTEH [5] and CPEB1 [6], and confirmed the prognostic value of these genes in astrocytomas.

LIM-only protein 3 (LMO3), a member of the LIM-only protein group, is a new DNA methylation gene that was identified in gliomas via the MeDIP-Chip [3]. The LIM domain is a zinc-binding amino acid motif that characterizes various proteins and functions in protein-protein interactions and transcriptional regulation. LIM-only proteins consist of 4 members: LMO1, LMO2, LMO3 and LMO4. LMO1, LMO2 and LMO3 express specificity in different cells of the adult mammalian central nervous system [7], and LMO1 and LMO2 act as oncogenes in acute T-cell lymphoblastic leukemia. LMO4 has been shown to be involved in the progression of breast cancer, and LMO3 acts as a molecular adaptor for protein-protein interactions. LMO3 can bind to the calcium- and integrin-binding protein CIB and induce C8 astrocyte proliferation [8]. Aoyama *et al*. reported that LMO3 is overexpressed and interacts with the neuronal transcription factor HEN2, inducing carcinogenesis in neuroblastomas [9, 10]. Additionally, the expression of LMO3 has been shown to be suppressed by DNA methylation in lung cancer [11].

MicroRNAs (miRNA) are ~22-nucleotide-long noncoding RNA molecules that usually function as endogenous repressors of target genes. MiRNAs can regulate multiple cellular processes, including proliferation, apoptosis, senescence, differentiation and development [12]. MiR-101 has been reported to be decreased and act as a tumor suppressor in various tumors, including osteosarcomas [13], cervical cancer [14], hepatocellular carcinomas [15], and breast cancer [16]. Our previous data demonstrated that miR-101 is also a tumor suppressor in gliomas and is a potential glioma marker [6].

The polycomb repressive complex 2 (PRC2) exerts oncogenic effects in many tumor types and silences gene expression by trimethylating histone 3 at lysine 27 (H3K27me3) [17]. Enhancer of zeste homolog 2 (EZH2) and embryonic ectoderm development (EED) encode components of PRC2 and have been found to play essential roles in cancer initiation, development, progression, metastasis, and drug resistance [18]. Previous studies have shown that EZH2 and EED are direct targets of miR-101 [19]. Downregulation of the trimethylated lysine 27 of histone H3 (H3K27me3) has been observed together with the upregulation of EZH2 following the re-expression of miR-101 in pancreatic ductal adenocarcinoma [20].

In this study, we confirmed the function of miR-101 in glioma cell apoptosis and demonstrated that miR-101 suppresses the expression of LMO3 through histone modification-induced hypomethylation.

## RESULTS

### LMO3 is overexpressed due to promoter hypomethylation and is correlated with a poor outcome in gliomas

**Figure 1:**
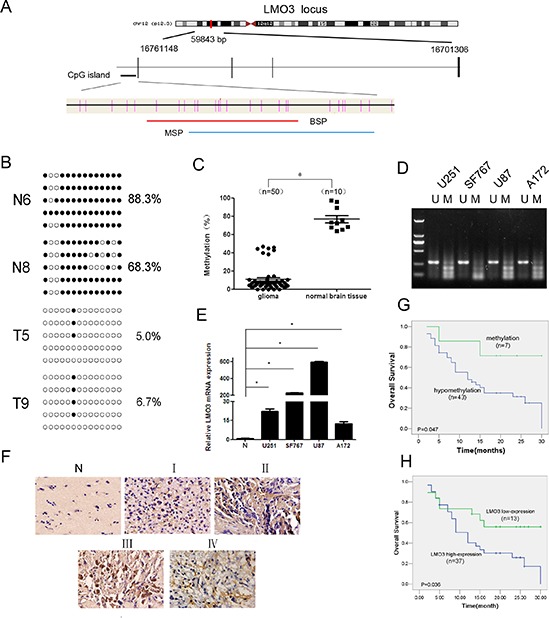
LMO3 is overexpressed due to promoter hypomethylation and is correlated with a poor outcome in gliomas **(A)** A schematic diagram of the CpG dinucleotides within the LMO3 promoter. The nucleotide number is relative to the transcription start site of LMO3. The red line indicates the region that was tested with BSP; the blue line indicates the region that was detected with MSP. **(B)** A BSP of the upstream regulatory region of LMO3 was performed for the representative tissues (N, normal brain tissue; T, glioma sample). For each sample, at least five separate clones were sequenced, and the results are shown here. The unmethylated CpG sites are shown as open circles, whereas the methylated CpG sites are indicated with closed circles. For each row of circles, the sequence results for an individual clone of the bisulfite-PCR product are given. The number of methylated CpGs divided by the total number of true CpGs analyzed is given as a percentage to the right of each BSP result. **(C)** The methylation status of LMO3 was detected using BSP in the glioma samples (*n* = 50) and normal brain tissues (*n* = 10). This was verified using an independent sample *t*-test. *p < 0.01. **(D)** The methylation status of LMO3 in the glioma cell lines was detected using MSP. U, unmethylated primer; M, methylated primer. **(E)** Real-time PCR was used to detect the expression of LMO3. The expression level of LMO3 in the normal brain tissue was much lower than in the four glioma cell lines. This was verified using an independent sample *t*-test. *p < 0.01. **(F)** The expression of LMO3 in normal brain tissues and glioma tissues was tested using ISH. **(G)** The correlation between the LMO3 methylation in the tumor tissues and the OS of the glioma patients. The patients with hypomethylation of LMO3 had a shorter OS than those with normal levels of LMO3 methylation. The Kaplan–Meier method was used. **(H)** The correlation between the expression of the LMO3 protein in the tumor and the OS of the glioma patients. The patients with a high level of LMO3 expression had a poor outcome. The Kaplan–Meier method was used.

We previously demonstrated that the LMO3 gene, located at 12p12.3, is hypomethylated in glioma tissues [3]. To further confirm the hypomethylation of LMO3 in gliomas, we designed and validated the BSP and MSP methods (Figure 1A). The CpG dinucleotides were heavily methylated in the normal brain samples of Cohort 1 (*n* = 4, 88.4 ± 9.9%) and Cohort 2 (*n* = 10, 76.9% ± 12.6%), whereas methylation was decreased in the glioma samples (Cohort 2, *n* = 50, 10.7% ± 13.6%; Cohort 1, *n* = 6, 40.1 ± 21.9%) (*p* < 0.05 or 0.001) (Figure 1B and Supplementary Figure S1). Forty-three of the 50 (86.0%) samples were hypomethylated (*p* < 0.001) (Figure 1C). A decrease in the methylation was observed in the glioma cell lines (Figure 1D). There were no statistically significant correlations between the sex, age or histological grade and LMO3 hypomethylation (Table 1). The expression of LMO3 in normal brain tissues was lower compared to the glioma cell lines (Figure 1E), and increased expression of the LMO3 protein was found in the glioma cell lines and in 37 of the 50 glioma tissues (Figure 1F). There were no statistical correlations between the sex, age or histological grade and the expression of LMO3 (Table 2). A comparison of the methylation status with the protein expression revealed that 34 of the 37 tumors that had a high level of LMO3 expression were hypomethylated (Table 1). There was a significant relationship between the hypomethylation of the LMO3 promoter and the overexpression of the LMO3 protein (χ2-test, *p* < 0.05, Table 1). The correlation between the LMO3 expression, methylation status and overall survival (OS) was statistically significant (Figure 1G and 1H). These results suggest that LMO3 overexpression and hypomethylation may be involved in glioma carcinogenesis, and LMO3 plays a potential role in glioma prognosis.

**Table 1:** Correlation between LMO3 methylation status, protein expression and clinical parameters of astrocytoma patients

Variable	LMO3		*P*
	hypomethylation	methylation	
Total (N = 50)	43	7	
Expression			0.043
<8 (13)	9	4	
≥8 (37)	34	3	
Sex			0.057
Male (34)	30	4	
Female (16)	13	3	
Age (years)^a^			0.857
<42 (27)	23	4	
≧42 (23)	20	3	
Grade			0.318
low grade (I+II) (26)	22	4	
high grade (III+IV) (24)	21	3	

a median age is 42 years;

Abbreviation: LMO3, LIM-only protein 3

**Table 2:** Correlation between LMO3 expression and clinical parameters of astrocytoma patients

Variable	LMO3		*P*
	< 8	≥ 8	
Total (N = 50)	13	37	
Sex			0.508
Male (34)	10	24	
Female (16)	3	13	
Age (years)^a^			0.526
<42 (27)	8	19	
≧42 (23)	5	18	
Grade			0.424
low grade (I+II) (26)	8	18	
high grade (III+IV) (24)	5	19	

a median age is 42 years;

### LMO3 is an epigenetic target of miR-101

**Figure 2:**
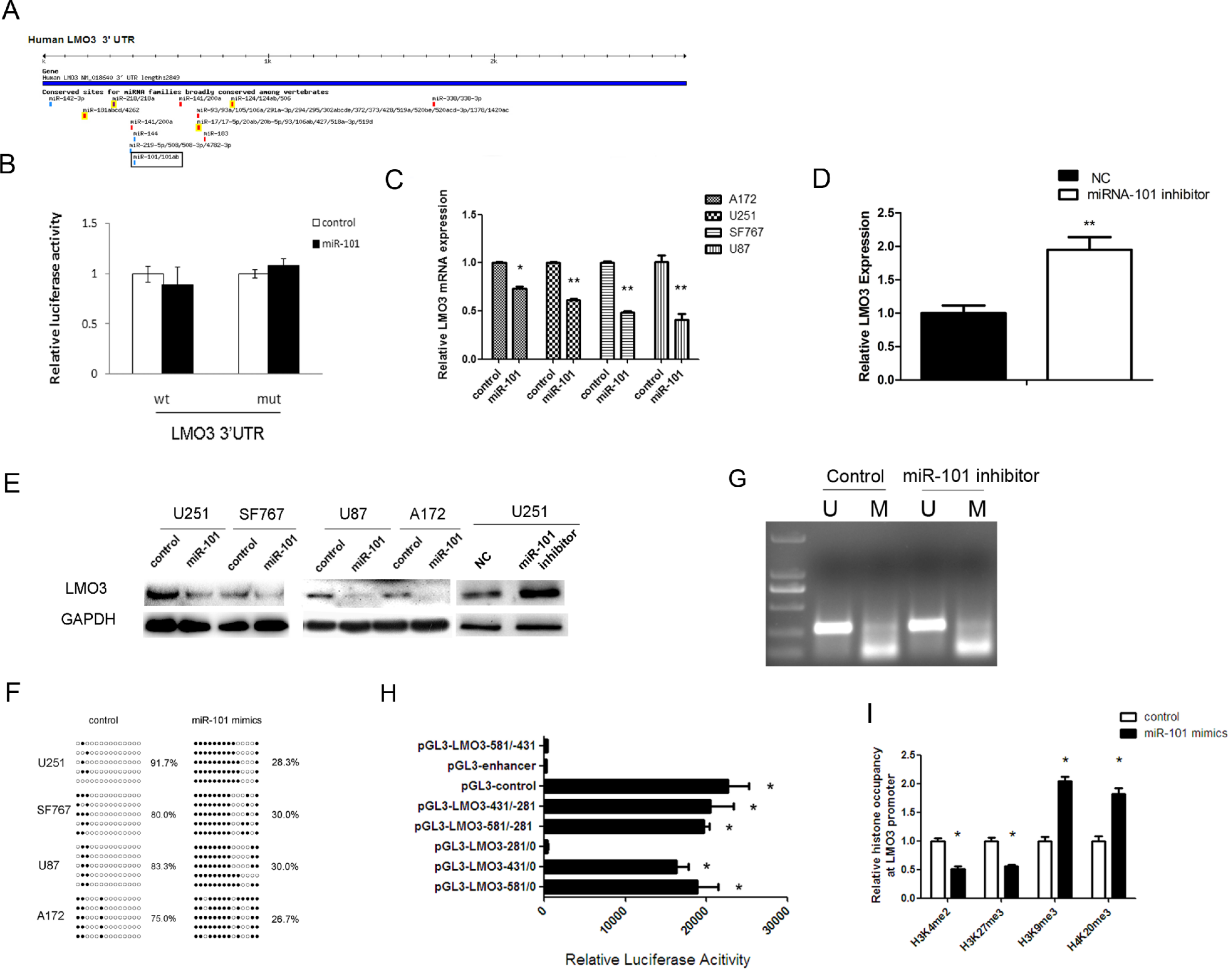
LMO3 is an epigenetic target gene of miR-101 **(A)** LMO3 was predicted to be a target gene of miR-101 using the online software program TargetScan 5.1. **(B)** MiR-101 did not regulate the expression of the CPEB1 3′-UTR reporter constructs. The luciferase reporter assays were performed 48 h after transfection with the indicated pMIR-REPORT plasmid and a Renilla transfection control plasmid, which were cotransfected with miR-101 or a relevant scrambled control. The data shown are the mean ± S.D. of six replicates and are representative of three independent experiments. An independent sample *t*-test was used. *p < 0.05. **(C)** MiR-101 inhibits the expression of LMO3 mRNA. A real-time PCR analysis was performed 48 h after transfection with miR-101 mimics and a scrambled control. An independent sample *t*-test was used. *p < 0.05. **p < 0.01. **(D)** MiR-101 knockdown enhances the expression of LMO3 mRNA. Real-time PCR analysis was performed 48 h after transfection with an inhibitor of miR-101 and a negative control. An independent sample *t*-test was used. **p < 0.01. **(E)** MiR-101 regulates the expression of the LMO3 protein. Western blot analysis was performed 72 h after transfection with miR-101 mimics, an inhibitor or a scrambled control. GAPDH was used as an internal control. **(F)** The methylation level of LMO3 was increased by miR-101 in U251 cells. The unmethylated and methylated CpG sites are indicated by open and closed circles, respectively. Each row indicates the sequencing result of one clone of the bisulfite-PCR product. The number of methylated CpGs was divided by the total number of true CpGs analyzed and is given as a percentage to the right of each BSP result. **(G)** An miR-101 inhibitor had no effect on the methylation status of LMO3, as determined via MSP in U251 cells. **(H)** An analysis of the promoter activity of the LMO3 core promoter constructs via luciferase reporter assays. The construct containing the sequence spanning the region from –431 to –281 was sufficient to mediate maximal promoter activity. The core promoter ranged from –431 to –281. PGL3-control is the positive control, and pGL3-enhancer is the negative control. An independent sample *t*-test was used. *p < 0.05. **(I)** The histone occupancy of the LMO3 promoter was affected by miR-101. A ChIP assay was used to detect the H3K4me2, H3K27me3, H3K9me3 and H4K20me3 occupancy of the LMO3 core promoter. U251 cells were transfected with miR-101 mimics or a scrambled control. An independent sample *t*-test was used. *p < 0.05.

Having established the hypomethylation role for LMO3 in gliomas, we next aimed to clarify the regulatory mechanism of LMO3 expression. We used the online software TargetScan 5.1 (Cambridge, MA, USA) to predict potential miRNA binding sites in the 3′-UTR sequence of LMO3. LMO3 was predicted to be a target of miR-101 (Figure 2A), and the predicted binding sites were cloned downstream of the firefly luciferase gene in the pMIR-REPORT vector (Supplementary Figure S2). When the cells were cotransfected with miR-101 and pMIR-LMO3– 3′-UTR-WT, there was no significant reduction in luciferase activity compared with cells transfected with the negative control (Figure 2B). Our previous study demonstrated the suppressor role of miR-101 in gliomas [6]; this finding is consistent with the results of the present study, which showed that the overexpression of miR-101 (Supplementary Figure S3A) inhibited the expression of LMO3 (Figure 2C and 2E) and that the knockdown of miR-101 (Supplementary Figure S3B) could enhance the expression of LMO3 in glioma cell lines (Figure 2D and 2E). LMO3 can therefore be considered to be a new indirect target of miR-101 in gliomas. Our previous study confirmed that miR-101 suppresses its targets via histone modification [6]. To determine whether miR-101 inhibits the expression of LMO3 via histone modification, the methylation status of LMO3 was detected using BSP and MSP. The demethylation rate was decreased in the glioma cell lines following the transfection with the miR-101 mimics (Figure 2F). However, the effect of the miR-101 inhibitor on the methylation status of the LMO3 promoter was not significant (Figure 2G). Subsequently, we determined the core promoter region of LMO3. The luciferase reporter assay demonstrated that the core promoter ranged from –431 to –281 (Figure 2H). As shown in Figure 2I, we found that the occupancy of H3K4me2 and H3K27me3 was decreased, whereas the occupancy of H3K9me3 and H4K20me3 was increased at the LMO3 core promoter in the miR-101 mimic-treated cells compared to the control. These results indicate that miR-101 inhibits the expression of LMO3 epigenetically in glioma cells.

### MiR-101 inverses the promoter methylation status of LMO3 through histone modification

**Figure 3:**
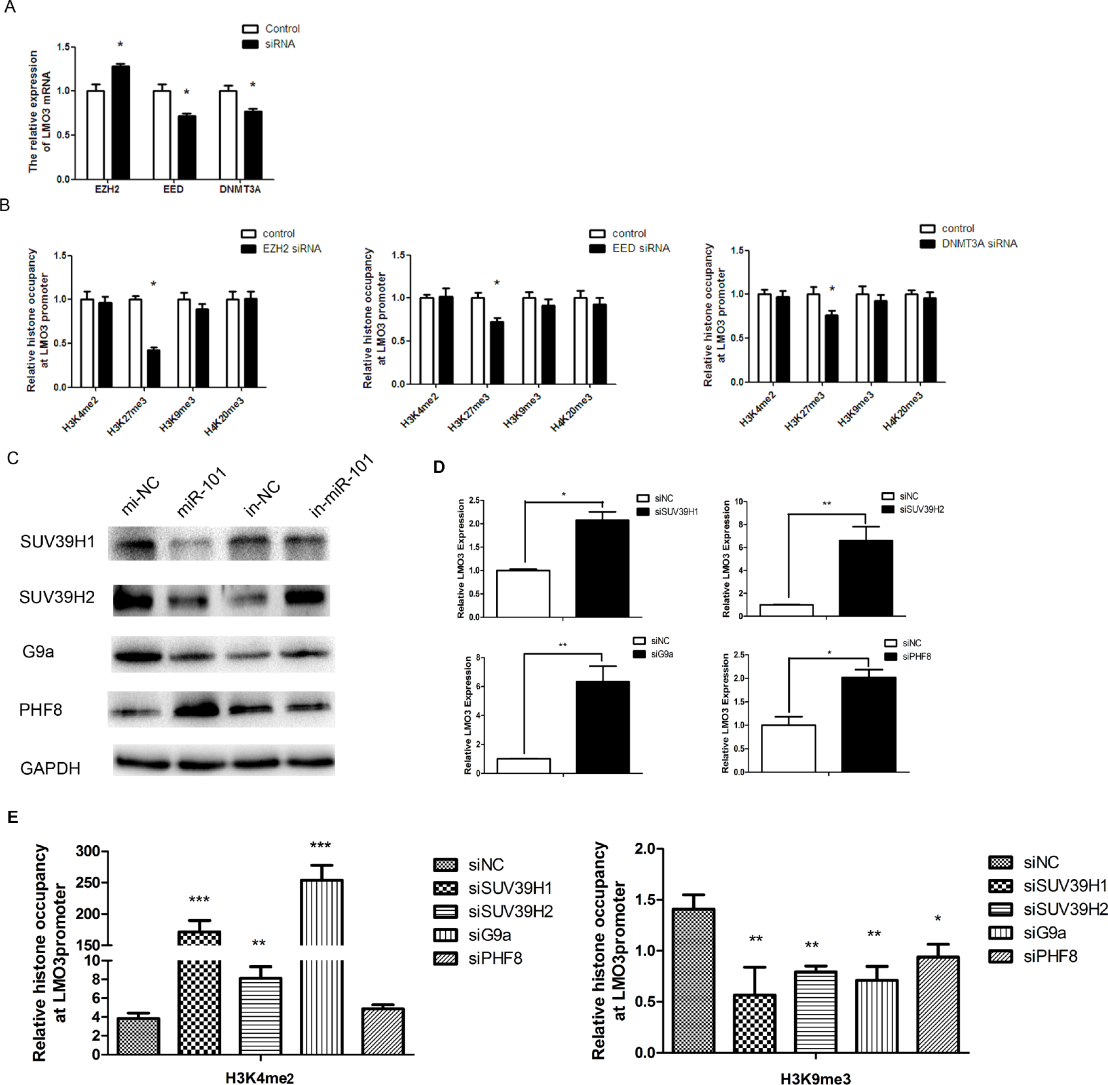
MiR-101 regulates the promoter methylation status of LMO3 through histone modification **(A)** The expression of LMO3 was regulated by EZH2, EED and DNMT3A. A real-time PCR analysis was performed 48 h after transfection with EZH2 siRNA, EED siRNA, DNMT3A siRNA or a scrambled control. An independent sample *t*-test was used. *p < 0.05. **(B)** The histone occupancy of the LMO3 promoter was affected by EZH2 siRNA, EED siRNA and DNMT3A siRNA. A ChIP assay was performed to detect the H3K4me2, H3K27me3, H3K9me3 and H4K20me3 occupancy of the LMO3 core promoter. U251 cells transfected with EZH2 siRNA, EED siRNA and DNMT3A siRNA were analyzed. An independent sample *t*-test was used. *p < 0.05. **(C)** MiR-101 regulated the expression of SUV39H1, SUV39H2, G9a and PHF8. Western blotting was performed 72 h after transfection with miR-101 mimics, an inhibitor or the negative control. GAPDH was used as the internal control. **(D)** The expression of LMO3 was regulated by SUV39H1, SUV39H2, G9a and PHF8A. A real-time PCR analysis was performed 48 h after transfection with SUV39H1 siRNA, SUV39H2 siRNA, G9a siRNA, PHF8 siRNA or a scrambled control. An independent sample *t*-test was used. *p < 0.05, **p < 0.01. **(E)** The histone occupancy of the LMO3 core promoter was affected by SUV39H1, SUV39H2, G9a and PHF8A. A ChIP assay was performed to detect the H3K9me3 occupancy of the LMO3 core promoter. U251 cells transfected with SUV39H1 siRNA, SUV39H2 siRNA, G9a siRNA and PHF8A siRNA were analyzed. An independent sample *t*-test was used. *p < 0.05. **p < 0.01.

Studies have shown that EZH2, EED and DNMT3A are direct targets of miR-101, and miR-101 decreases the expression of EZH2, EED and DNMT3A [19]. When EZH2 was inhibited (Supplementary Figure S4, upper line), the expression of LMO3 increased. When EED or DNMT3A were knocked down in U251 cells (Supplementary Figure S4, upper line), the expression of LMO3 decreased (Figure 3A). The occupancy of the methylation-related histones (H3K4me2, H3K27me3, H3K9me3 and H4K20me3) on the LMO3 promoter was detected after the inhibition of EZH2, EED and DNMT3A in glioma cells. Only the H3K27me3 occupancy was decreased by the inhibition of EZH2, EED and DNMT3A (Figure 3B). Subsequently, we determined whether miR-101 could regulate the expression of SUV39H1, SUV39H2, G9a and PHF8. When miR-101 was overexpressed, the expression of SUV39H1, SUV39H2 and G9a decreased, while PHF8 increased. Additionally, the expression of SUV39H1, SUV39H2 and G9a was stimulated, and the expression of PHF8 was suppressed by the miR-101 inhibitor (Figure 3C). We then determined that the suppression of SUV39H1, SUV39H2, G9a and PHF8 (Supplementary Figure S4, lower line) could enhance the expression of LMO3 (Figure 3D). The occupancy of H3K9me3 was decreased by the inhibition of SUV39H1, SUV39H2, G9a or PHF8, as detected by ChIP (Figure 3E).

### MiR-101 inhibits the binding of USF and myeloid zinc finger 1 (MZF1) to the LMO3 promoter

**Figure 4:**
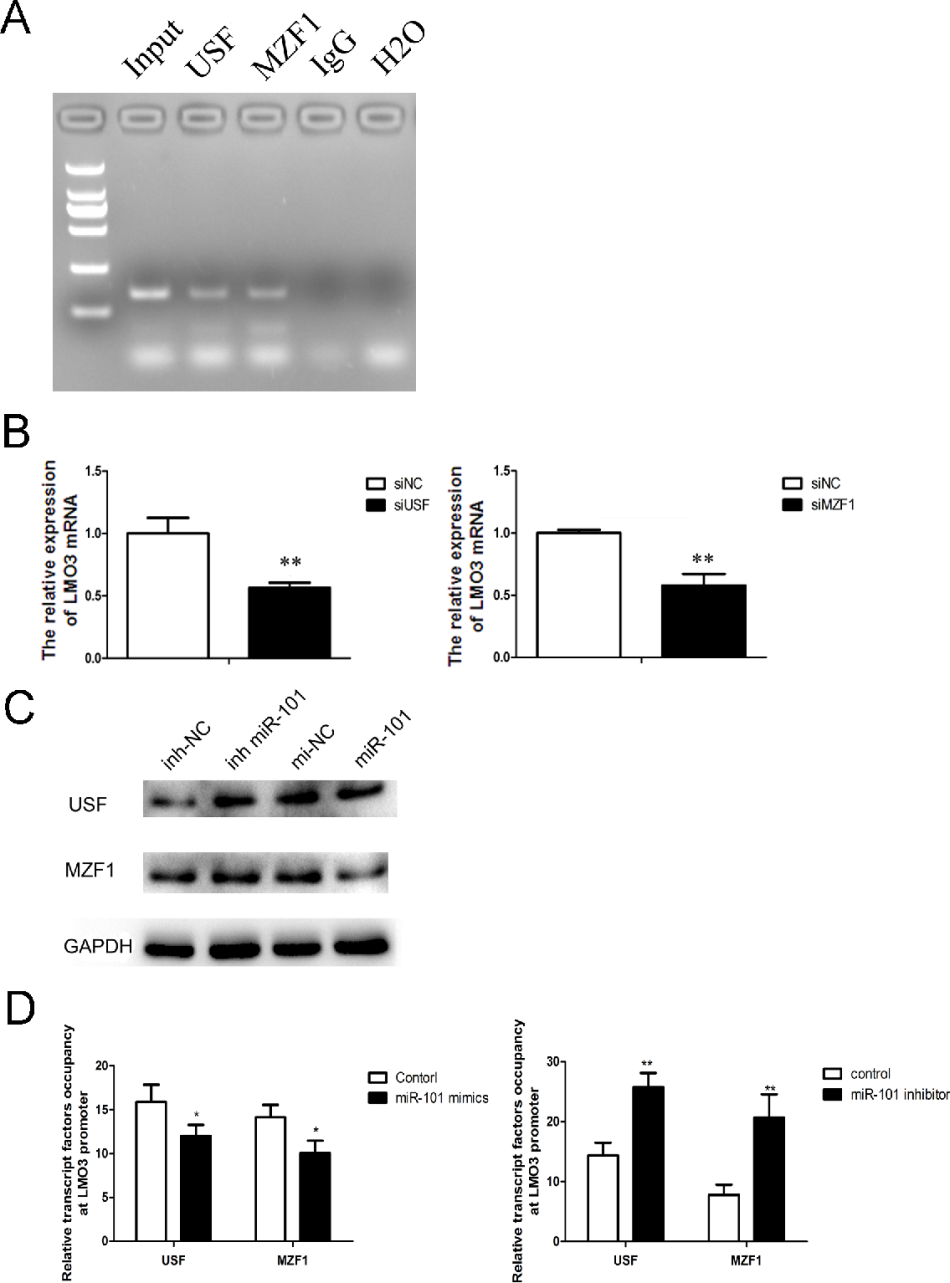
MiR-101 suppresses the binding of USF and MZF1 to the LMO3 promoter **(A)** USF and MZF1 bound to the LMO3 promoter. A ChIP assay was performed to detect the binding of USF and MZF1 to the LMO3 promoter in U251 cells. Normal mouse IgG was used as a negative control. **(B)** The expression of LMO3 was regulated by USF and MZF1. A real-time PCR analysis was performed 48 h after transfection with USF siRNA, MZF1 siRNA or a scrambled control. An independent sample *t*-test was used. **p < 0.01. **(C)** MiR-101 regulated the expression of USF and MZF1. Western blotting was performed 72 h after transfection with miR-101 mimics, miR-101 inhibitor or a negative control. GAPDH was used as an internal control. **(D)** The transcription factor occupancy of the LMO3 promoter was affected by miR-101. A ChIP assay was performed to evaluate the USF and MZF1 occupancy of the LMO3 core promoter. Left: U251 cells transfected with miR-101 mimics and a negative control. Right: U251 cells transfected with a miR-101 inhibitor and a negative control. An independent sample *t*-test was used. *p < 0.05. **p < 0.01.

To identify the mechanism of the miR-101 regulation of LMO3, we predicted the potential transcription factors on the LMO3 promoter using TFSEARCH ver. 1.3. The results indicated that USF and MZF1 bind to the LMO3 promoter, which was verified via the ChIP assay (Figure 4A). As shown in Figure 4B, the expression of LMO3 was decreased by the inhibition of USF and MZF1 (Supplementary Figure S4, lower line). Furthermore, overexpression of miR-101 inhibited the expression of USF and MZF1, and the inhibition of miR-101 enhanced the expression of USF and MZF1 (Figure 4C). The effects of miR-101 on the binding of USF and MZF1 to the LMO3 promoter was detected via the ChIP assay. The data showed that the overexpression of miR-101 disrupted the binding of USF and MZF1 to the LMO3 promoter (Figure 4D). These results illustrate that miR-101 inhibits the binding of USF and MZF1 to the LMO3 promoter and inhibits the expression of LMO3.

### MiR-101 induces the apoptosis of glioblastoma cells by suppressing LMO3

**Figure 5:**
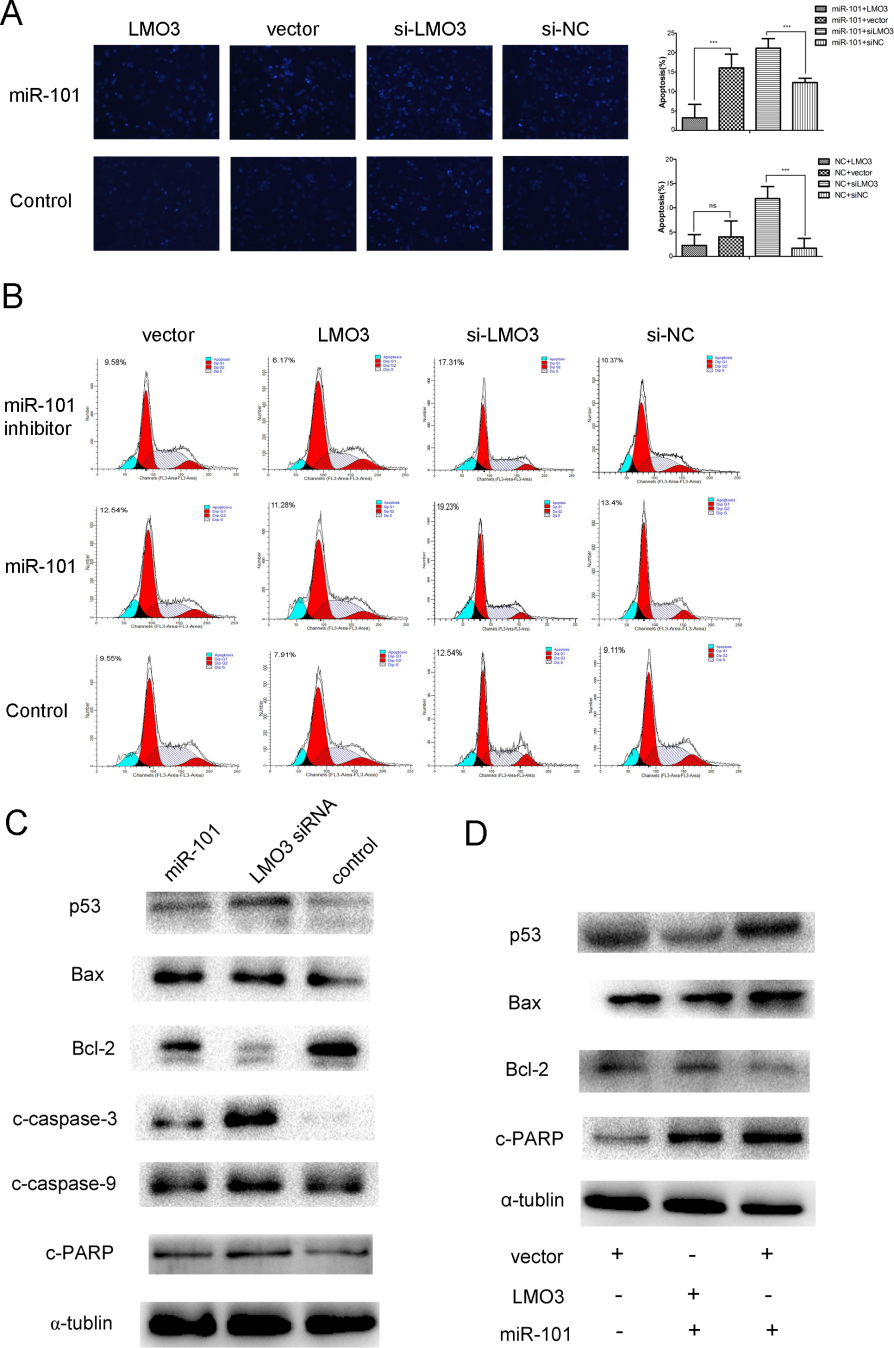
MiR-101 induces U251 cell apoptosis via LMO3 **(A)** LMO3 overexpression counteracted miR-101-promoted apoptosis (×100). Left: Hoechst 33258 staining was used to detect the level of apoptosis in U251 cells. Right: The bar graph represents the apoptosis cells counts. The U251 cells were first transfected with miR-101 mimics and NC (control) and then with pcDNA3.1-LMO3 or empty vector pcDNA3.1, LMO3 siRNA or a scrambled control. An independent sample *t*-test was used. **p < 0.01. **(B)** The apoptosis of the U251 cells was detected using flow cytometry. The U251 cells were first transfected with miR-101 mimics or NC and were then transfected with pcDNA3.1-LMO3 or empty vector pcDNA3.1, LMO3 siRNA or a scrambled control. **(C)** Both miR-101 and LMO3 regulated the expression of apoptosis-related molecules in U251 cells. Western blotting was performed to detect the expression of p53, Bax, Bcl-2, caspase-3, caspase-9, and c-PARP. The U251 cells were transfected with miR-101 mimics, LMO3 siRNA or a scrambled control; α-tubulin was used as an internal control. **(D)** The overexpression of LMO3 abrogated the expression of apoptosis-related molecules regulated by miR-101. Western blotting was performed to detect the expression of p53, Bax, Bcl-2 and c-PARP; α-tubulin was used as an internal control.

In this study, the ectopic overexpression of miR-101 was able to increase the level of apoptosis in the U251 cells, as detected via Hoechst staining and FCM. This effect, which is induced by miR-101 overexpression, could be disrupted by LMO3 overexpression (Figure 5A and 5B). The overexpression of miR-101 or the inhibition of LMO3 increased the expression of p53, Bax, cleaved caspase-3, cleaved caspase-9 and cleaved PARP and inhibited the expression of Bcl-2 (Figure 5C) in the U251 cells. The overexpression of LMO3 was able to interrupt the upregulation of p53, Bax and cleaved PARP and the downregulation of Bcl-2 induced by miR-101 (Figure 5D). These results suggest that miR-101 is able to induced apoptosis by targeting LMO3.

## DISCUSSION

Changes in the methylation status of gene promoters are generally accepted to indicate a poor prognosis in gliomas [23]. We identified that hypomethylation is one of the mechanisms of LMO3 overexpression in astrocytomas. Using the Kaplan–Meier method, we found that astrocytoma patients who had overexpression or hypomethylation of LMO3 had poor outcomes. Therefore, LMO3 overexpression and hypomethylation can be considered to be potential markers for the prognosis of astrocytomas.

It has been reported that LMO3 is a transcriptional regulator involved in central nervous system development [24], lung adenocarcinomas [25] and neuroblastomas [10]. In lung cancer, LMO3 was detected to be hypomethylated [11]. In neuroblastomas, the increased expression of LMO3 contributes to the tumor development and aggressiveness. LMO3 acts as a co-repressor of p53, suppressing p53-dependent transcriptional regulation without inhibiting its DNA-binding activity [26]. In this study, the inhibition of LMO3 could induce apoptosis by upregulating the expression of the caspase signal pathway and the Bcl-2 family in U251 cells.

Aside from hypomethylation, the mechanisms of LMO3 dysregulation in gliomas is unclear. We first predicted the miRNAs that could bind to the 3′-UTR of LMO3. MiR-101 was predicted to bind to the 3′-UTR of LMO3. However, we identified that miR-101 inhibits the expression of LMO3 not by binding to its 3′-UTR but in an indirect way in glioma cells. MiR-101 has been reported to be a tumor suppressor in multiple cancers, such as thyroid cancer [27], hepatomas [28], breast cancer [16], and endometrial cancer [12]. One of the mechanisms by which miR-101 suppresses tumors is the induction of apoptosis [16]. MiR-101 sensitizes the A549 NSCLC cell line to CDDP by activating caspase 3-dependent apoptosis [29]. In our study, miR-101 was shown to induce glioma cell apoptosis by indirectly inhibiting LMO3.

**Figure 6:**
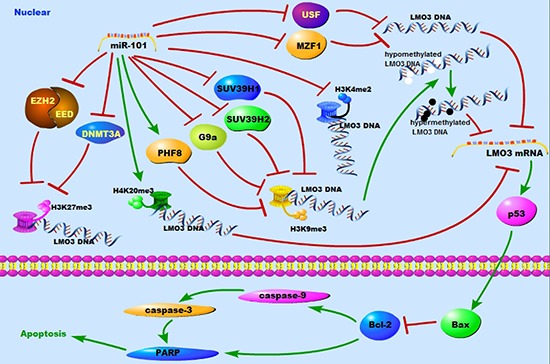
A schematic diagram of the working model for the miR-101 targeting system in glioma cells

The mechanism by which miR-101 inhibits the expression of LMO3 is unknown. It is well known that EZH2 [12] and DNMT3A [30] are targets of miR-101. EZH2 is a driver of H3K27 trimethylation [31], and DNMT3A stimulates DNA methylation [32]. Therefore, we hypothesized that miR-101 suppresses the expression of LMO3 in a histone- or methylation-associated pattern. Over the past several years, a series of studies have identified methylated K4, K9, and K27 on histone H3. Di- or trimethylation of H3K4 activates the gene's promoter. Di- or trimethylation of H3K27 and H4K20 represses the gene's promoter. Although the dimethylation of H3K9 represses the gene's promoter, the trimethylation of H3K9 can activate or repress the promoter [2]. In this study, we found that the occupancy of H3K4me2 and H3K27me3 was decreased by miR-101, whereas the occupancy of H3K9me3 and H4K20me3 was increased at the LMO3 core promoter in glioma cells. Additionally, only the H3K27me3 occupancy was decreased by the inhibition of EZH2, EED and DNMT3A. This indicates that miR-101 may regulate the occupancy of H3K4me2, H3K9me3 and H4K20me3 via their specific histone methyltransferases. KDM5B is reported to modify the methylation status of H3K4 [33], whereas SUV39H1/2 [34] and G9a [35] mediate the trimethylation of H3K9. G9a is a specific histone methyltransferase of H3K9 and inhibits the self-renewal of glioma cancer stem cells [36]. PHF8 removes repressive histone markers, including H4K20me1 and H3K9me1/2, and binds to H3K4me3 [37]. We identified that miR-101 inhibits the expression of SUV39H1/2 and G9a but enhances the expression of PHF8 in glioma cells. We also showed that miR-101 decreases the occupancy of H3K27 by inhibiting EZH2, DNMT3A and EED. MiR-101 was found to decrease the H3K9me3 occupancy on the LMO3 promoter by inhibiting SUV39H1, SUV39H2, G9a and PHF8. Although serial studies have shown that G9a acts as an oncogene, the overexpression of G9a suppresses sphere formation in glioma cancer stem cells [36]. Therefore, G9a may play a different role in gliomas, and there may be other mechanisms of miR-101 that regulate H4K20me3 in glioma cells (Figure 6).

USF and MZF1 are transcription factors. The inhibition of USF protects against oxygen- and glucose-deprivation-induced apoptosis via the downregulation of miR-132 in HepG2 cells [38]. The binding of USF-1 to an E-box of promoter can up-regulate the expression of cathepsin B and TGF-beta2 in glioma cells [39, 40]. MZF1 promotes the progression of multiple cancers, including colorectal cancer [41], lung adenocarcinomas [42] and cervical cancer [43]. There are currently no data showing the function of MZF1 in gliomas. In this study, we first verified that USF and MZF1 were transcription factors that bind to the LMO3 promoter. We then demonstrated that miR-101 could suppress the expression of LMO3 by decreasing the expression USF and MZF1 and interrupting their binding to the LMO3 promoter (Figure 6).

In this study, we identified the following: LMO3 is increased in glioma cell lines and tissues and is correlated with a poor prognosis; miR-101 induces apoptosis by suppressing LMO3; and miR-101 inhibits the expression of LMO3 by recovering its methylation status via the regulaton of methylated histone occupation and the suppression of the transcription factors USF and MZF1 in gliomas.

## MATERIALS AND METHODS

### Tissue specimens

We obtained frozen tissue samples of 50 gliomas and 10 normal brain tissues from the Xiangya Hospital of Central South University, Hunan, China, between January 2009 and July 2011. The study was approved by the Ethical Committee of the Faculty of Medicine, Central South University, and informed consent was obtained from all of the patients. The tumor samples were diagnosed using the World Health Organization system by two pathologists who were blinded to the patient data. Clinical data, including the gender, age, initial presentation, postoperative irradiation, chemotherapy, follow-up and outcome, were obtained from medical records. The samples included 16 female and 34 male patients ranging from 16 to 65 years old, with a mean age of 41 years and a median age of 42 years [6].

### Cell lines and treatments

The following human glioma cell lines obtained from the Cell Center of Peking Union Medical College (Beijing, China) were used: U251, A172, SHG44 and U87. The U251, A172 and U87 cells were maintained in Dulbecco's Modified Eagle medium (Gibco, Grand Island, NY, USA), and the SHG44 cells were maintained in RPMI-1640 (Gibco) with 10% FCS, 100 units/ml of penicillin and 100 mg/ml of streptomycin at 37°C in a humidified atmosphere of 5% CO2 and 95% air.

### Isolation of genomic DNA from cell lines and tissues and bisulfite DNA treatment

Genomic DNA was isolated from the cell lines, glioma tissues and normal brain tissues using the Universal Genomic DNA Extraction Kit Ver. 3.0 (Takara, Dalian, China) according to the manufacturer's instructions. The genomic DNA (0.5 mg) that was extracted from the cells, tumors and the normal tissue specimens was subjected to bisulfite treatment using an Epitect Bisulfite Kit (Qiagen, Hilden, Germany) and stored at –20°C until further use.

### Bisulfite sequencing PCR and methylation-specific PCR

BSP and MSP were conducted as described previously [21], beginning with the amplification of the bisulfite-treated LMO3 promoter containing 14 CpG sites. For PCR, 2.5 U of Taq mix (Takara) and 0.5 μl of 1 mM forward and reverse primers were used in a 50-μl total reaction volume. A total of 100 ng of bisulfite-treated DNA was then used as the template for PCR. The PCR cycles were as follows: 95°C for 5 min, followed by 38 cycles at 95°C for 0.5 min, 61.7°C for 2 min and 72°C for 2.5 min, followed by a final extension at 72°C for 10 min. The PCR products were purified via gel extraction from a 1% agarose gel and ligated into the pGEM-T vector (Promega, Madison, WI, USA) using a 3:1 vector/PCR product ratio. The ligation products were used to transform competent *Escherichia coli* cells (strain JM109) using standard procedures, and blue/white screening was used to select a minimum of five bacterial transformants (clones). The LMO3 promoter of the positive clones was sequenced by Genscript (Nanjing, China) and Invitrogen (Guangzhou, China). The decrease in the methylation for each sample was calculated as the percent of unmethylated CpG dinucleotides from the total number of CpG dinucleotides that were analyzed. For the MSP, 2.5 U of Taq mix (Takara) and 0.5 μl of 1 mM forward and reverse primers were used in a 25-μl total reaction volume. Subsequently, 50 ng of bisulfite-treated DNA was used as the template for PCR. The PCR cycles were as follows: 95°C for 5 min, followed by 35 cycles at 95°C for 0.5 min, 60°C for 2 min and at 72°C for 1 min, followed by a final extension at 72°C for 10 min. The PCR products were separated on 1% agarose gels and analyzed via Sybrsafe staining.

### MicroRNAs, siRNAs, DNA plasmids and transfection

MiR-101 mimics and their associated control and the miR-101 inhibitors and their associated control were synthesized by GenePharma Co., Ltd. (Shanghai, China). To generate a luciferase reporter construct, we synthesized 54 bp from the 3′-UTR of the LMO3 mRNA from human genomic DNA (Invitrogen). This 3′-UTR region of LMO3, which contains the predicted target sites for miR-101, was then subcloned downstream of the pMIR-REPORT miRNA expression reporter vector (Ambion, Shanghai, China). We also constructed plasmids with mutated miR-101 target sites. The miR-101 mimics, siRNA and DNA plasmids were transfected using Lipofectamine 2000 (Life Technologies, Gaithersburg, MD, USA).

### Cloning of the LMO3 promoter, plasmid construction and transfection

Different upstream regulatory regions of the LMO3 gene were amplified from U251 DNA using PCR with UltraPF DNA polymerase (GeneCopoeia Inc., Rockville, MD, USA). The PCR fragments were digested with MluI/XhoI and linked to the luciferase-based promoter-less plasmid-pGL3-Enhancer Vector (Promega) to create the following plasmids: pGL3-LMO3–581/0, pGL3-LMO3–431/0, pGL3-LMO3–281/0, pGL3-LMO3–581/-281, pGL3-LMO3–431/-281, and pGL3-LMO3–581/-431. The sequences and the orientation of the cloned fragments were confirmed by direct DNA sequencing. The plasmids used for transfection were isolated and purified using the Purelink Plasmid Mini 25 Reaction Kit (Invitrogen–Life Technologies, Carlsbad, CA, USA). The promoter activities of these fragments were tested via the transient transfection of 1 mg of plasmid DNA into the U251 cell lines using Lipofectamine2000. For the luciferase-based assay, the results were normalized against the Renilla luciferase activity. At least three-independent assays were performed.

### Luciferase reporter assay

U251 cells were plated in a 24-well plate and then cotransfected with 0.5 nmol of either the miR-101 mimics or a scrambled control, 20 ng of either pMIR-LMO3–3′-UTR-WT or pMIR-LMO3–3′-UTR-MUT and 2 ng of pRL-TK (Promega). Cells were collected 48 h after the transfection and analyzed using the Dual-Luciferase Reporter Assay System (Promega). Luciferase activity was detected using an M200 microplate fluorescence reader (Tecan, Beijing, China). The pMIR-REPORT-β-gal control vector was cotransfected as an internal control to correct for differences in both the transfection and harvest efficiencies. The transfection experiments were performed in duplicate and repeated in at least three independent experiments.

### RNA extraction, reverse transcription and real-time quantitative PCR

Total RNA was extracted from glioma cells using Trizol (Life Technologies, Rockville, MD, USA). For real-time PCR, 2 mg of the total RNA was reverse-transcribed using a cDNA synthesis kit (Fermentas, Burlington, ON, Canada). The β-actin gene was used as a control for this reaction. The data were normalized to β-actin levels, and the level of LMO3 mRNA in the glioma cell lines was determined using the 2^−ΔΔCt^ method. The miR-101 levels were determined using a SYBR-green-containing PCR kit (GenePharma Co.), and the RNA input was normalized to the level of human U6 snRNA.

### Western blotting

The cell protein lysates, cytosol proteins and nuclear proteins were separated on 10% SDS-polyacrylamide gels, electrophoretically transferred to polyvinylidene difluoride membranes (Millipore, Danvers, MA, USA), and detected using a goat polyclonal antibody for LMO3 (Santa Cruz Biotechnology, Santa Cruz County, CA, USA), a mouse monoclonal antibody for EZH2, EED (Santa Cruz Biotechnology) and GAPDH (Millipore), and a rabbit polyclonal antibody for H3K27me3, H3K9me3, H4K20me3 and H3K4me2 (Millipore) using a commercial ECL kit (Pierce, Rockford, IL, USA). The intensity of the protein fragments was quantified using ChemicalDocTM XRSþ (Bio-Rad, Berkeley, CA, USA).

### Hoechst staining

To morphologically detect the level of apoptosis, treated or untreated cells were cultured for 48 h, fixed with 4% paraformaldehyde, and stained with Hoechst 33258 (0.5 μg/ml) for 15 min. The cells were observed using a microscope and recorded. The nuclei of the dead cells were stained by the Hoechst stain, and the number of dead cells was recorded.

### Flow cytometry

Cells were cultured in each well of a 6-well plate and transfected with miR-101 mimics, miR-101 inhibitor, LMO3 siRNA, LMO3-pcDNA3.1 or a scrambled sequence. Cell apoptosis was then assessed 48 h after the transfection. The cells were washed three times using cold PBS and were then fixed in 70% ethanol and stored at 4°C for subsequent cell cycle analyses. After fixation, the cells were stained using PI (Beyotime, Beijing, China) and analyzed using a flow cytometer (Beckman).

### Immunohistochemical staining

The immunohistochemical studies were performed using the UltraSensitive SP Kit (Maixin Biotechnology Company, Fuzhou, China), and the color was developed using DAB as the chromogen. The cells were then counterstained with Mayer's hematoxylin and mounted for microscopic evaluation (OLYMPUS BX-51 microscope, Osaka, Japan). Two independent pathologists who were blinded to the clinicopathological information performed the scoring. The staining was scored to determining the intensity (1, 0+; 2, 1+; 3, 2+; 4, 3+) and the percent of membranous and cytoplasmic staining in the malignant cells (1, 0–25%; 2, 26–50%; 3, 51–75%; 4, 76–100%). The intensity score multiplied by the percentage counts was used as the final score. A score of < 8 was considered to be low-level expression, whereas a score > 8 was considered to be high-level expression.

### ChIP assay and qRT-PCR

A total of 2 × 10^7^ cells of each parental U251 line were used for the ChIP assay. The PCR-ChIP analysis was performed as previously described [22]. The chromatin was incubated at 4°C overnight with antibodies specific to dimethylated H3K4, trimethylated H3K9, trimethylated H3K27 and trimethyl-H4K20 (Millipore); normal mouse or rabbit IgG was used as the control. Real-time PCR amplification was performed the Power SYBR Green PCR Master Mix according to the manufacturer's instructions (Takara). Immunoprecipitated DNA from three independent ChIP analyses with the anti-H3K4me2, anti-H3K9me3, anti-H3K27me3 and anti-H4K20me3 antibodies was subjected to a real-time quantitative PCR performed in triplicate. The data are presented as the ‘Relative Occupancy’, which was calculated using the equation 2 ^[ΔCt (No antibody–Input)−ΔCt (Target–Input)]^.

### Follow-up study

In this prospective study, the patients were followed up for a period of up to 2.5 years. None of the patients received radiation or chemotherapy before their surgery. Postoperatively, the patients were subjected to a physical examination every 3 months for 1 year and a brain CT examination every 6 months thereafter. The overall survival, defined as the time from the date of surgery to the date of death or last contact if the patient was still alive ranged from 2 to 30 months (median, 13 months). Among the 50 glioma patients included in this study, a complete follow-up was available for 38 patients. Fifteen patients who did not show recurrence were alive until the end of the follow-up period. Thirty-two patients died due to disease recurrence.

### Statistical analysis

Data are presented as the mean ± S.D. from at least three separate experiments. Multiple group comparisons were performed using ANOVA with a post hoc test for the subsequent individual group comparisons. The difference in the LMO3 promoter methylation status between the normal brain tissues and the glioma tissues was examined using an independent sample *t*-test. The relationships between the LMO3 methylation status, protein expression and clinicopathological parameters were examined using the χ^2^-test. The OS curves were calculated using the Kaplan–Meier method, and the log-rank test was used to determine the difference in the OS rates between the two groups. The results were considered to be significant when *a* value of *p* < 0.05 was obtained. All of the statistical analyses were performed using SPSS13.0 for Windows (SPSS Inc., Chicago, IL, USA).

## SUPPLEMENTARY FIGURES


