# Correction: MiR-101 reverses the hypomethylation of the LMO3 promoter in glioma cells

**DOI:** 10.18632/oncotarget.27716

**Published:** 2020-12-08

**Authors:** Xiaoping Liu, Qianqian Lei, Zhibin Yu, Gang Xu, Hailin Tang, Wei Wang, Zeyou Wang, Guiyuan Li, Minghua Wu

**Affiliations:** ^1^ Hunan Provincial Tumor Hospital and the Affiliated Tumor Hospital of Xiangya Medical School, Central South University, Changsha 410013, Hunan, China; ^2^ Department of Breast Oncology, Sun Yat-Sen University Cancer Center, State Key Laboratory of Oncology in South China, Collaborative Innovation Center for Cancer Medicine, Guangzhou 510060, Guangdong, China; ^3^ School of Basic Medical Science, Cancer Research Institute, Central South University, Key Laboratory of Carcinogenesis and Cancer Invasion, Ministry of Education, Key Laboratory of Carcinogenesis, Ministry of Health, Changsha 410078, Hunan, China; ^4^ Medical College, University of South China, Hengyang 421001, Hunan, China

**This article has been corrected:** During assembly of Figure 5A, the authors made an error in the vector and scramble sequences co-transfected group, whose picture was the same as the si-NC and scramble sequences co-transfected group. The corrected Figure 5A is shown below. The authors declare that these corrections do not change the results or conclusions of this paper.

Original article: Oncotarget. 2015; 6:7930–7943. 7930-7943. https://doi.org/10.18632/oncotarget.3181

**Figure 5:**
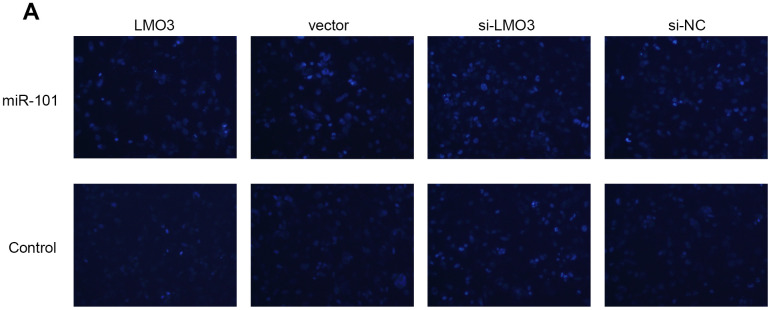
MiR-101 induces U251 cell apoptosis via LMO3. (**A**) LMO3 overexpression counteracted miR-101-promoted apoptosis (×100). Left: Hoechst 33258 staining was used to detect the level of apoptosis in U251 cells.

