# Supplementary material for: MiR-101 reverses the hypomethylation of the LMO3 promoter in glioma cells
Source: Oncotarget. 2015 Feb 11;6(10):7930–43. doi: 10.18632/oncotarget.3181 (PMC4480726; doi:10.18632/oncotarget.3181)
Supplement: Supplementary file 1 [file oncotarget-06-7930-s001.pdf]

SUPPLEMENTARY FIGURES

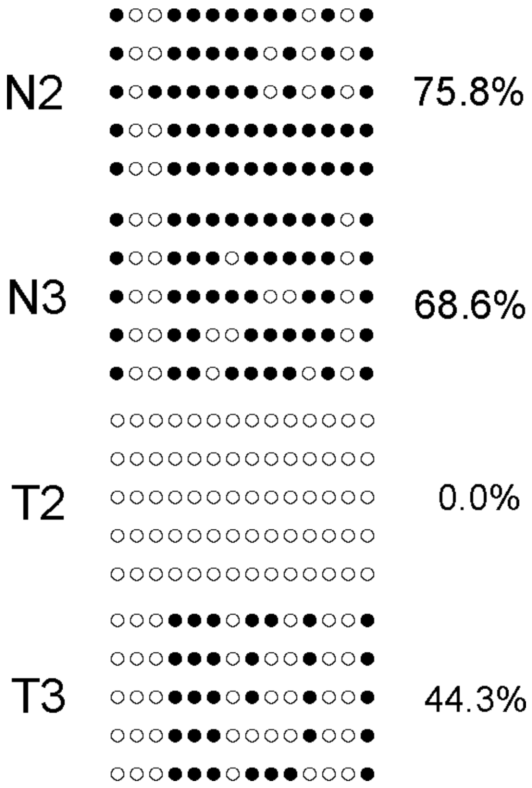

Supplementary Figure S1: LMO3 promoter methylation was analyzed by BSP in normal brain tissue (N2, N3) and astrocytoma sample (T2, T3). For each sample, at least five separate clones were sequenced. Black and white dots indicate methylation and unmethylation of a CpG , respectively. The number of methylated CpGs divided by the total number of true CpGs analyzed is given as a percentage on the righ to feach BSP result.

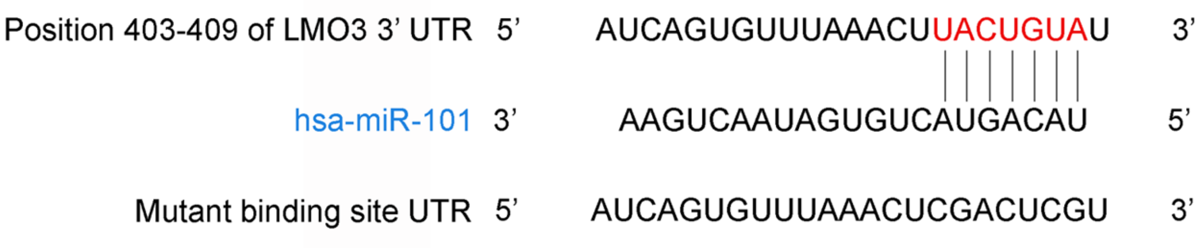

Supplementary Figure S2: The miR-101 targeting site in LMO3 3'-UTR is shown. Wild type and the mutated form of LMO3 3'-UTR are shown.

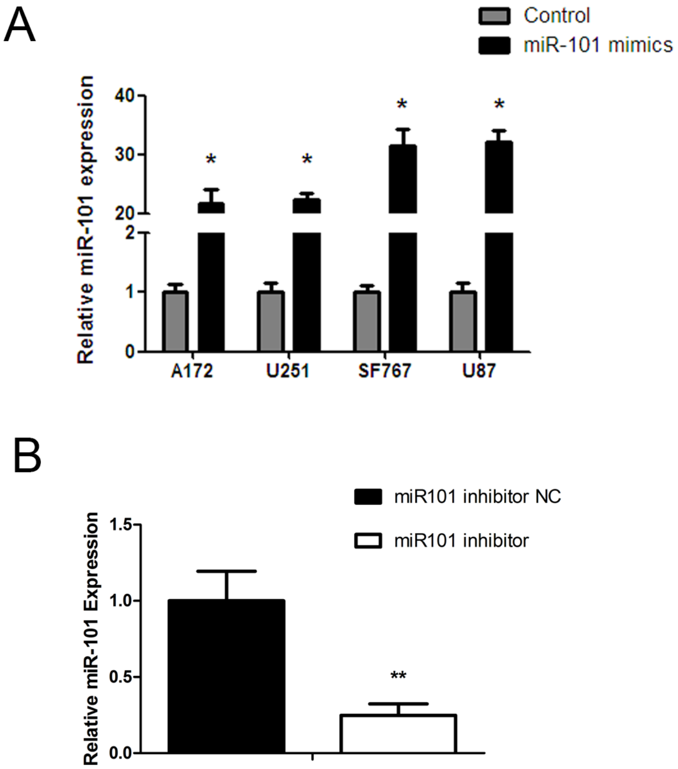

Supplementary Figure S3: The transfection efficiency of miR-101 mimics or miR-101 inhibitor was detected in glioma cells. (A) The transfection efficiency of miR-101 mimics was performed in A172, U251, SF767 and U87 cells. *$P < 0.05$. **(B)** The transfection efficiency of miR-101 inhibitor was performed in U251 cells. **$P < 0.01$.

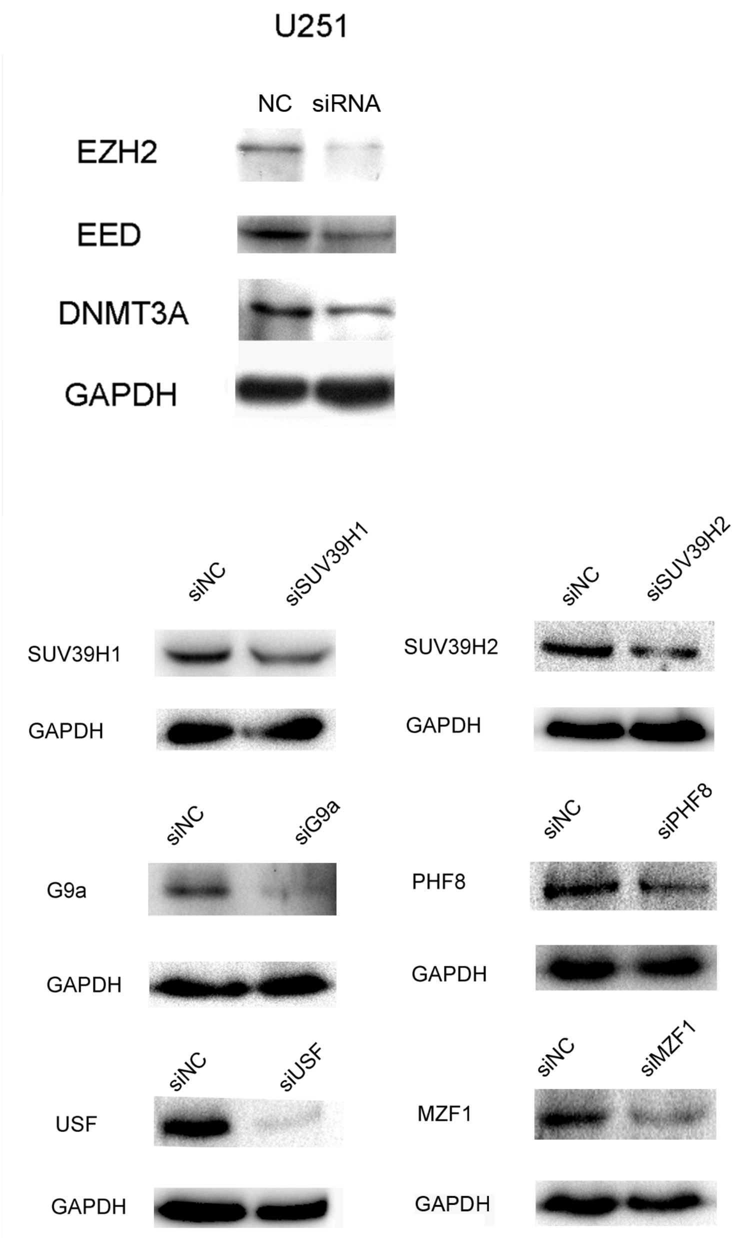

Supplementary Figure S4: The transfection efficiency of siRNAs were detected in U251 cells. Western blot were used to monitor the expression of endogenous EZH2, EED, DNMT3A, SUV39H1, SUV39H2, G9a, PHF8, USF and MZF1 at 72h after transfection with siRNA or control RNA.
